# Supplementary material for: Characterization of an African Swine Fever Virus Field Isolate from Vietnam with Deletions in the Left Variable Multigene Family Region
Source: Viruses. 2024 Apr 7;16(4):571. doi: 10.3390/v16040571 (PMC11054794; doi:10.3390/v16040571)
Supplement: Supplementary file 1 [file viruses-16-00571-s001.zip › viruses-2923252-supplementary.pdf]

ASFV Georgia 2007/1 - Dilution	MGF 360-14L	Tignon
Undiluted	25.51	25.63
10^{-1}	28.54	28.93
10^{-2}	31.44	32.51
10^{-3}	35.84	36.94
10^{-4}	37.45	-
10^{-5}	-	-
10^{-6}	-	-
NTC	-	-

Table S1. Sensitivity of MGF 360-14L real-time PCR compared to that of Tignon real-time PCR assay. Total nucleic acid extracted from cell culture amplified ASFV Georgia 2007/1 ten-fold dilutions, was tested by the MGF 360-14L and Tignon real-time PCR assays. 2007/1.

Sample	MGF 360-14L	Tignon	ß-Actin
DPI 9 P1	-	25.71	20.37
DPI 9 P2	-	31.69	22.30
DPI 9 P3	-	32.47	20.06
DPI 9 P4	-	35.36	22.36
DPI 9 P5	-	-	19.82
DPI 9 P6	-	35.28	21.92
DPC 0 P2	-	38.84	25.47
DPC 0 P3	-	-	23.86
DPC 0 P4	-	38.50	25.34
DPC 0 P5	-	38.45	24.53
DPC 0 P6	-	-	24.20
DPC 4 P2	-	38.43	25.23
DPC 4 P3	-	-	22.59
DPC 4 P4	-	-	22.48
DPC 4 P5	-	-	24.40
DPC 4 P6	-	-	24.21
DPC 7 P2	-	-	24.19
DPC 7 P3	-	-	24.16
DPC 7 P4	-	-	23.27
DPC 7 P5	-	39.32	22.34
DPC 7 P6	-	-	23.60
DPC 15 P2	-	-	21.67
DPC 15 P3	-	39.20	21.19
DPC 15 P4	-	-	21.48
DPC 15 P5	-	-	20.30
DPC 15 P6	-	-	21.42
DPC 21 P2	-	-	21.69
DPC 21 P3	-	-	21.67
DPC 21 P4	-	-	21.40
DPC 21 P5	-	37.38	21.12
DPC 21 P6	-		21.87
DPC 6 P7	18.47	20.34	23.71
EC	-	-	-
NTC	-	-	-
ASFV-GUS-Vietnam Inoculum	-	24.25	31.26
ASFV Georgia 2007/1	25.41	26.19	30.81

Table S2. Detection of ASFV MGF 360-14L in whole blood samples collected from pigs ON inoculated with ASFV-GUS-Vietnam and challenged with ASFV Georgia 2007/1 IM. DPI= days post infections with ASFV-GUS-Vietnam. Tignon RT-PCR detects p72 gene that is present in both ASFV Georgia 2007/1 and ASFV-GUS-Vietnam. ASFV MGF 360-14L is present only in ASFV Georgia 2007/1. EC= extraction control. NTC= non-template control. DPI= days post infection. . DPC= days post challenge.

Sample	MGF 360-14L	Tignon	ß-Actin
DPI 9 P1	-	33.70	24.54
DPI 9 P2	-	33.09	25.32
DPI 9 P3	-	39.17	26.46
DPI 9 P4	-	37.46	29.29
DPI 9 P5	-	-	27.50
DPI 9 P6	-	33.33	29.44
DPC 0 P2	-	-	29.71
DPC 0 P3	-	-	28.94
DPC 0 P4	-	38.11	29.41
DPC 0 P5	-	-	29.14
DPC 0 P6	-	-	32.36
DPC 4 P2	-	-	30.01
DPC 4 P3	-	-	28.83
DPC 4 P4	-	-	28.73
DPC 4 P5	-	-	30.27
DPC 4 P6	-	-	29.55
DPC 7 P2	-	38.00	30.43
DPC 7 P3	-	38.22	30.09
DPC 7 P4	33.54*	36.15	28.42
DPC 7 P5	-	-	29.69
DPC 7 P6	-	-	30.40
DPC 15 P2	-	-	28.33
DPC 15 P3	-	-	28.16
DPC 15 P4	-	33.04	28.68
DPC 15 P5	-	-	30.65
DPC 15 P6	-	-	29.18
DPC 21 P2	-	-	29.97
DPC 21 P3	-	-	28.12
DPC 21 P4	-	36.08	28.57
DPC 21 P5	-	-	28.71
DPC 21 P6	-	-	29.46
DPC 4 P7	26.13	26.60	28.94
EC	-	-	-
NTC	-	-	-
ASFV- GUS-Vietnam Inoculum	-	22.36	30.25
ASFV Georgia 2007/1	25.18	24.79	28.73

Table S3. Detection of ASFV MGF 360-14L in OPSW samples collected from pigs ON inoculated with ASFV-GUS-Vietnam and challenged IM with ASFV Georgia 2007/1. Tignon RT-PCR detects p72 gene that is present in both ASFV Georgia 2007/ and ASFV-GUS-Vietnam. ASFV MGF 360-14L is present only in ASFV Georgia 2007/1. * Low levels of was detected in one OPSW sample. EC= extraction control. NTC= non-template control. DPI= days post infection. . DPC= days post challenge.

Sample	MGF 360-14L	Tignon	ß-Actin
Spleen P1*	-	19.09	22.06
Spleen P2	-	-	21.45
Spleen P3	-	38.32	20.33
Spleen P4	-	-	21.15
Spleen P5	-	-	21.03
Spleen P6	-	-	19.76
SILN P1*	-	27.08	23.50
SILN P2	-	-	32.27
SILN P3	-	-	26.23
SILN P4	-	37.21	27.24
SILN P5	-	35.72	22.22
SILN P6	-	-	24.26
EC	-	-	-
NTC	-	-	-
ASFV-GUS-Vietnam Inoculum	-	23.74	31.36
ASFV Georgia 2007/1	25.21	25.75	30.10

Table S4. Detection of ASFV MGF 360-14L in lymphoid tissues collected from pigs ON inoculated with ASFV-GUS-Vietnam and challenged IM with ASFV Georgia 2007/1. Tignon RT-PCR detects p72 gene that is present in both ASFV Georgia 2007/ and ASFV-GUS-Vietnam. ASFV MGF 360-14L is present only in ASFV Georgia 2007/1. EC= extraction control. NTC= non-template control. SILN = superficial inguinal lymph node. DPI= days post infection. DPC= days post challenge. Note= * P1 that succumbed to ASF shows the highest viral load in spleen and SILN.

Sample	MGF	Tignon	ß-Actin
DPI 9 P9	-	28.20	20.47
DPI 9 P10	-	38.13	21.73
DPI 9 P11	-	32.98	19.71
DPI 9 P12	-	28.16	20.42
DPI 9 P13	-	-	22.09
DPI 9 P14	-	-	20.69
DPC -1 P9	-	34.58	21.55
DPC -1 P10	-	-	20.24
DPC -1 P11	-	38.35	20.04
DPC -1 P12	-	37.95	19.99
DPC -1 P13	-	36.20	21.22
DPC 4 P9	-	35.00	22.77
DPC 4 P10	-	-	22.04
DPC 4 P11	-	-	21.69
DPC 4 P12	-	37.15	21.26
DPC 4 P13	-	35.94	22.32
DPC 7 P9	-	36.67	20.45
DPC 7 P10	-	-	21.81
DPC 7 P11	-	-	22.07
DPC 7 P12	-	-	21.88
DPC 7 P13	-	34.58	21.51
DPC 14 P9	-	34.72	20.76
DPC 14 P10	-	-	21.90
DPC 14 P11	-	-	22.52
DPC 14 P12	-	37.08	22.37
DPC 14 P13	-	34.78	21.42
DPC 25 P9	-	36.15	21.00
DPC 25 P10	-	-	22.50
DPC 25 P11	-	-	22.38
DPC 25 P12	-	38.42	23.19
DPC 25 P13	-	-	22.48
EC	-	-	-
NTC	-	-	-
ASFV-Gus-Vietnam Inoculum	-	24.19	31.16
ASFV Georgia 2007/1	24.72	25.56	30.11

Table S5. Detection of ASFV MGF 360-14L in whole blood collected from pigs IM inoculated with ASFV-GUS-Vietnam and challenged ON with ASFV Georgia 2007/1. Tignon RT-PCR detects p72 gene that is present in both ASFV Georgia 2007/ and ASFV-GUS-Vietnam. ASFV MGF 360-14L is present only in ASFV Georgia 2007/1. EC= extraction control. NTC= non-template control. DPI= days post infection. DPC= days post challenge.

Samples	MGF 360-14L	Tignon	ß-Actin
DPI 13 P9	-	25.09	28.20
DPI 13 P10	-	37.15	29.77
DPI 13 P11	-	32.61	29.18
DPI 13 P12	-	27.87	29.62
DPI 13 P13	-	36.10	29.03
DPI 13 P14	-	33.97	27.94
DPC -1 P9	-	-	29.10
DPC -1 P10	-	-	28.79
DPC -1 P11	-	29.38	28.66
DPC -1 P12	-	-	28.37
DPC -1 P13	-	24.01	28.45
DPC 4 P9	-	-	27.97
DPC 4 P10	-	-	30.66
DPC 4 P11	-	31.46	27.86
DPC 4 P12	-	34.13	28.22
DPC 4 P13	-	26.53	27.62
DPC 7 P9	-	-	25.58
DPC 7 P10	-	-	29.09
DPC 7 P11	-	30.86	25.37
DPC 7 P12	-	-	28.20
DPC 7 P13	-	28.02	27.69
DPC 14 P9	-	-	30.05
DPC 14 P10	-	-	28.97
DPC 14 P11	-	33.54	27.75
DPC 14 P12	-	-	29.33
DPC 14 P13	-	29.53	28.98
DPC 25 P9	-	-	29.52
DPC 25 P10	-	-	28.72
DPC 25 P11	-	30.17	26.15
DPC 25 P12	-	-	27.54
DPC 25 P13	-	-	35.48
EC	-	-	-
NTC	-	-	-
ASF Georgia 2007/1	25.46	25.52	31.04
ASF GUS Vietnam Inoculum	-	27.97	33.29

Table S6. **Detection of ASFV MGF 360-14L in OPSW samples collected from pigs IM inoculated with ASFV-GUS-Vietnam and challenged ON with ASFV Georgia 2007/1.** Tignon RT-PCR detects p72 gene that is present in both ASFV Georgia 2007/ and ASFV-GUS-Vietnam. ASFV MGF 360-14L is present only in ASFV Georgia 2007/1. EC= extraction control. NTC= non-template control. DPI= days post infection. DPC= days post challenge.

	MGF 360-14L	Tignon	ß- actin
Spleen P9	-	36.07	17.54
Spleen P10	-	-	17.87
Spleen P11	-	-	19.83
Spleen P12	-	-	19.85
Spleen P13	-	38.51	19.32
Spleen P14	-	19.40	19.31
SILN P9	-	-	23.97
SILN P10	-	-	23.19
SILN P11	-	-	21.21
SILN P12	-	35.14	22.79
SILN P13	-	38.17	21.06
SILN P14	-	21.21	20.03
Tonsil P11	-	31.68	21.36
Tonsil P13	-	32.51	21.26
Tonsil P14	-	28.43	22.44
SMLN P11	-	33.23	21.52
SMLN P14	-	26.57	20.96
GHLN P12	-	35.6	21.57
GHLN p13	-	28.97	21.89
GHLN P14	-	22.22	21.97
EC	-	-	-
NTC	-	-	-
ASF Georgia 2007/1	22.84	23.37	28.32
ASFV- GUS-Vietnam Inoculum	-	22.25	29.26

Table S7. Detection of ASFV MGF 360-14L in lymphoid tissues collected from pigs IM inoculated with ASFV-GUS-Vietnam and challenged ON with ASFV Georgia 2007/1. Tignon RT-PCR detected p72 gene that is present in both ASFV Georgia 2007/1 and ASFV-GUS-Vietnam. ASFV MGF 360-14L is present only in ASFV Georgia 2007/1. EC = extraction control. NTC= non-template control. DPI = days post-infection. SMLN = sub-mandibular lymph node. GHLN = gastro-hepatic lymph node. SILN = superficial inguinal lymph node. Note: Only Tignon assay positive tonsil, SMLN, and GHLN were tested by MGF 360-14L RT-PCR. P14 = the contact pig that succumbed to ASF.

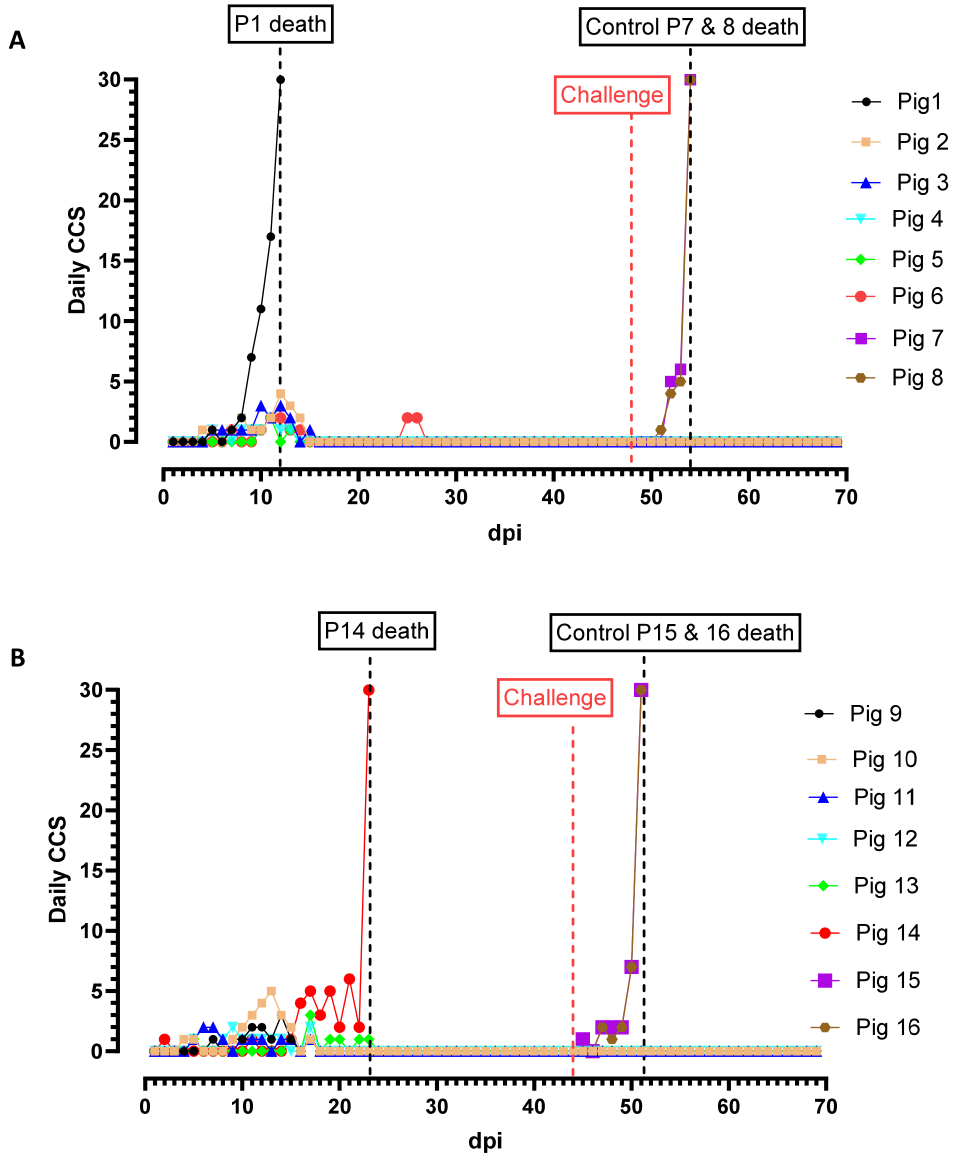

Figure S1: Daily cumulative clinical scores (CCS) of the pig # 1-8 (A) and pig # 9-16 (B). Pig # 1-6 were inoculated with ASFV-GUS-Vietnam ON followed by ASFV Georgia 2007/1 challenge IM. The breed and age matched control pigs (# 7 and 8) were challenged with ASFV Georgia 2007/1 IM. Pigs # 9 – 14 were inoculated with ASFV-GUS-Vietnam virus IM and challenged with ASFV Georgia 2007/1 ON. The breed and age matched control pig # 15 and 16, were challenged with ASFV Georgia 2007/1 ON. When a pig reached the humane end point or found dead, the highest possible CCS of 30 was assigned. dpi = days post-infection
